# Development and validation of a nomogram integrating inflammatory-nutritional-metabolic composite indices for predicting short-term adverse outcomes in hospitalized patients with pneumonia

**DOI:** 10.3389/fmed.2026.1846682

**Published:** 2026-06-01

**Authors:** Jing Zhao, Tao Bao

**Affiliations:** Department of Respiratory and Critical Care Medicine, Yixing People's Hospital, Yixing, China

**Keywords:** inflammatory-nutritional-metabolic indices, nomogram, pneumonia, prognostic prediction, risk stratification

## Abstract

**Objective:**

To develop and validate a prediction model integrating multidimensional inflammatory-nutritional-metabolic composite indices for early risk stratification of short-term adverse outcomes in hospitalized pneumonia patients.

**Methods:**

A total of 839 hospitalized pneumonia patients admitted between January 2020 and December 2023 were retrospectively enrolled and randomly divided into a training cohort (*n* = 587) and a validation cohort (*n* = 252) at a 7:3 ratio. The primary endpoint was in-hospital intensive care unit (ICU) admission and/or invasive mechanical ventilation (IMV). Independent predictors were selected using least absolute shrinkage and selection operator (LASSO) regression combined with multivariable logistic regression, and a nomogram was constructed accordingly. Random Forest and XGBoost machine learning models were additionally built to corroborate variable importance. Model performance was evaluated using the area under the receiver operating characteristic curve (AUC), calibration curves, decision curve analysis (DCA), and net reclassification improvement (NRI)/integrated discrimination improvement (IDI). A simplified integer-based THM score was subsequently derived from the three highest-weighted inflammatory-nutritional-metabolic indices.

**Results:**

The overall incidence of short-term adverse outcomes was 17.6% (148/839). Following LASSO selection and multivariable analysis, nine independent predictors were identified: monocyte-to-high-density lipoprotein cholesterol ratio (MHR), triglyceride-glucose (TyG) index, neutrophil-to-lymphocyte ratio (NLR), hemoglobin-albumin-lymphocyte-platelet (HALP) index, peripheral oxygen saturation (SpO₂), partial pressure of arterial oxygen to fraction of inspired oxygen ratio (PaO₂/FiO₂), CURB-65 score, procalcitonin (PCT), and age. The nomogram achieved AUCs of 0.871 and 0.848 in the training and validation cohorts, respectively, significantly outperforming the Pneumonia Severity Index (PSI; AUC = 0.752) and CURB-65 (AUC = 0.728) (both *p* < 0.05). The Hosmer-Lemeshow (H-L) test confirmed satisfactory calibration (validation cohort *p* = 0.621), and DCA demonstrated superior net clinical benefit over traditional scores across a wide range of threshold probabilities. NRI and IDI analyses confirmed significant incremental predictive value over both reference tools (all *p* < 0.001). The THM simplified score (AUC = 0.804) stratified patients into low-risk (4.7%), intermediate-risk (16.6%), and high-risk (25.2%) groups, with a significant gradient trend (*p* < 0.001).

**Conclusion:**

The nomogram and THM simplified score integrating inflammatory-nutritional-metabolic composite indices significantly outperform conventional PSI and CURB-65 in predicting short-term adverse outcomes among hospitalized pneumonia patients, providing practical quantitative tools for early precise triage and individualized clinical decision-making.

## Introduction

Pneumonia remains one of the leading infectious causes of hospitalization and mortality worldwide, placing a persistent burden on public health systems (1). Despite widespread availability of antimicrobial agents in modern clinical practice, critically ill pneumonia patients continue to face substantial rates of in-hospital complications and mortality, with a considerable proportion requiring admission to the intensive care unit (ICU) or life-sustaining interventions such as invasive mechanical ventilation (IMV) (2, 3). Consequently, accurate early risk stratification and prognostic assessment represent central challenges in guiding clinical decision-making and optimizing allocation of healthcare resources (4, 5).

Currently, the Pneumonia Severity Index (PSI) and CURB-65 score are the most widely used prognostic tools for pneumonia and are endorsed by several international guidelines for directing hospitalization and treatment-intensity decisions (6, 7). However, these traditional scoring systems carry inherent limitations: the PSI encompasses numerous variables and involves a complex calculation process that is impractical for rapid assessment in the emergency department or at the bedside (6); CURB-65, while more straightforward, is constructed from a limited set of physiological and functional parameters and fails to adequately capture the multidimensional advances in understanding pneumonia pathophysiology, leaving substantial room for improvement in predictive accuracy—particularly in identifying high-risk individuals (8, 9).

Accumulating evidence indicates that infection-induced systemic inflammatory responses, host nutritional status, and metabolic disturbances play critical roles in the pathological progression and clinical outcomes of pneumonia. The neutrophil-to-lymphocyte ratio (NLR), a classic inflammatory marker, reflects the degree of inflammatory activation and immune imbalance (10–12). The hemoglobin-albumin-lymphocyte-platelet (HALP) index integrates inflammation, nutritional status, and immune function into a single quantitative measure, and has demonstrated independent prognostic value across various critical illnesses (13–15). The triglyceride-glucose (TyG) index serves as a surrogate marker of insulin resistance; recent investigations suggest that metabolic dysfunction is closely associated with adverse outcomes in infectious diseases (16, 17). The monocyte-to-high-density lipoprotein cholesterol ratio (MHR) integrates pro-inflammatory signaling mediated by monocytes with the anti-inflammatory and antioxidative properties of high-density lipoprotein cholesterol (HDL-C), also exhibiting potential prognostic utility (18, 19). Recent evidence has further extended this paradigm to respiratory infection: the prognostic nutritional index has been shown to independently stratify mortality risk in ICU-admitted community-acquired pneumonia (20), and integrative inflammatory-biomarker frameworks have demonstrated incremental prognostic value over conventional severity scores in critical illness cohorts (21)—collectively reinforcing the rationale for systematically incorporating multidimensional composite indices into pneumonia-specific prognostic modeling. Although these inflammatory-nutritional-metabolic composite indices have been extensively investigated in cardiovascular disease, sepsis, and oncology, their combined predictive value for short-term adverse outcomes in hospitalized pneumonia patients has not been systematically evaluated. Furthermore, existing studies have largely failed to conduct head-to-head comparisons between multidimensional composite indices and traditional scoring systems with rigorous internal validation.

In light of the above, this study aimed to systematically integrate multidimensional inflammatory-nutritional-metabolic composite indices and, through least absolute shrinkage and selection operator (LASSO) regression combined with multivariable logistic regression, develop a visualized nomogram for predicting short-term adverse outcomes (ICU admission and/or IMV) in hospitalized pneumonia patients. Model discrimination, calibration, and clinical utility were systematically validated in an independent validation cohort using a comprehensive evaluation framework comprising receiver operating characteristic (ROC) curves, calibration curves, decision curve analysis (DCA), and net reclassification improvement (NRI)/integrated discrimination improvement (IDI) analyses, with assessment of incremental predictive value over PSI and CURB-65. Furthermore, to meet the practical demands of rapid bedside assessment, a simplified integer THM score integrating the TyG index, MHR, and HALP index was derived from the multivariable model, aiming to provide a more practical tool for early precise triage and individualized clinical decision-making in pneumonia patients.

## Methods

### Study design and participants

This was a retrospective, study approved by the Ethics Committee of Yixing People’s Hospital. Informed consent was waived due to the observational nature of our retrospective analysis. Consecutive hospitalized pneumonia patients admitted to the Respiratory Department between January 2020 and December 2023 were enrolled. Inclusion criteria were: (1) age ≥18 years; (2) clinical diagnosis of pneumonia based on newly developed pulmonary infiltrates accompanied by fever, cough, expectoration, or chest pain; (3) hospital stay ≥24 h; and (4) completion of relevant laboratory tests and vital signs documentation within 24 h of admission. Exclusion criteria were: (1) receipt of IMV or ICU admission at the time of hospital entry; (2) active pulmonary tuberculosis, lung abscess, or acute infectious exacerbation of bronchiectasis; (3) severe immunocompromise (including HIV infection, solid organ or bone marrow transplant recipients, or receipt of immunosuppressive therapy within the preceding 3 months); (4) missing critical clinical data exceeding 20%; and (5) in-hospital death from clearly non-pneumonia causes (e.g., major trauma, end-stage malignancy in documented terminal trajectory, primary cardiac or aortic catastrophe, or care withdrawal per advance directive), independently adjudicated by two senior respiratory physicians with disagreements resolved by a third; deaths in which sepsis, ARDS, or MODS were etiologically linked to the index pneumonia were retained. The study was approved by the institutional ethics committee; informed consent was waived given the retrospective nature of the study.

### Data collection

Demographic characteristics, comorbidity history, admission vital signs, and laboratory values were extracted from the electronic medical record system. Demographics included age, sex, body mass index (BMI), smoking history, and chronic alcohol use. Comorbidities recorded encompassed chronic obstructive pulmonary disease (COPD), diabetes mellitus, heart failure, coronary artery disease, chronic kidney disease, malignancy, cerebrovascular disease, and bronchial asthma. Vital signs at admission included heart rate, respiratory rate, systolic and diastolic blood pressure, body temperature, and peripheral oxygen saturation (SpO₂). Laboratory parameters were extracted from the first measurement within 24 h of admission, including complete blood count (white blood cell count, neutrophil count, lymphocyte count, monocyte count, eosinophil count, platelet count, and hemoglobin), inflammatory markers (C-reactive protein [CRP], procalcitonin [PCT], interleukin-6 [IL-6], and ferritin) (22, 23), nutritional indices (albumin, prealbumin, and transferrin), metabolic and lipid parameters (fasting glucose, triglycerides, high-density lipoprotein cholesterol [HDL-C], and low-density lipoprotein cholesterol [LDL-C]), hepatorenal and coagulation indices (creatinine, blood urea nitrogen [BUN], D-dimer, and fibrinogen), cardiac biomarkers (N-terminal pro-B-type natriuretic peptide [NT-proBNP] and cardiac troponin I [cTnI]), and the oxygenation index (PaO₂/FiO₂) derived from arterial blood gas analysis. PSI and CURB-65 scores were calculated as reference standards for traditional predictive models.

Of 882 consecutively screened patients, 43 were excluded prior to analysis: 22 for >20% missing critical data, 14 for IMV or ICU admission at hospital entry, and 7 for clearly non-pneumonia–related in-hospital death, yielding the final analytic cohort of 839. Among retained patients, item-wise missingness was low (median 1.4%, maximum 4.8% for transferrin and prealbumin); Little’s MCAR test was non-significant (*p* = 0.218), and missing values were handled by multiple imputation via chained equations (MICE; m = 20 imputations, predictive mean matching for continuous variables and logistic regression for binary variables) prior to LASSO selection, with downstream estimates pooled by Rubin’s rules. A complete-case sensitivity analysis yielded an essentially identical predictor set and *Δ* area under the receiver operating characteristic curve (AUC) < 0.005 versus the imputed primary analysis, indicating robustness to the missingness mechanism.

### Calculation of composite inflammatory-nutritional-metabolic indices

The NLR was defined as the ratio of neutrophil count to lymphocyte count. The HALP index was calculated as: HALP = hemoglobin (g/L) × albumin (g/L) × lymphocyte count (×10^9^/L)/platelet count (×10^9^/L). The TyG index was calculated as: TyG = ln[triglycerides (mg/dL) × fasting glucose (mg/dL)/2]. The MHR was defined as the ratio of monocyte count (×10^9^/L) to HDL-C (mmol/L). Additionally, the platelet-to-lymphocyte ratio (PLR), lymphocyte-to-monocyte ratio (LMR), and eosinophil-to-lymphocyte ratio (ELR) were computed as candidate variables.

### Outcome definition

The primary endpoint was a composite of short-term adverse in-hospital outcomes, defined as the occurrence of at least one of the following: (1) ICU admission; or (2) requirement for IMV. Secondary endpoints included total length of hospital stay, incidence of in-hospital complications (sepsis, septic shock, acute respiratory distress syndrome [ARDS], and acute kidney injury [AKI]), and 30-day readmission rate.

### Cohort splitting

Patients were randomly allocated to the training cohort (*n* = 587) and validation cohort (*n* = 252) at a 7:3 ratio using stratified random sampling with a fixed random seed. The stratification variable was the primary endpoint, ensuring balanced rates of adverse outcomes between the two groups. The training cohort was used for variable selection, model development, and parameter estimation; the validation cohort served for independent internal validation.

### Candidate variable selection: LASSO regression

To identify key predictors associated with the outcome from 25 candidate variables while controlling for overfitting, LASSO regression was applied to the training cohort. Ten-fold cross-validation was used to determine the optimal penalty parameter *λ*; the value of λ corresponding to the minimum cross-validation error (λ.min) was selected. Variables with non-zero coefficients after LASSO shrinkage were retained for subsequent multivariable analysis.

### Multivariable logistic regression and nomogram

Variables identified by LASSO regression were entered as independent variables, with the composite short-term adverse outcome as the dependent variable, into a multivariable logistic regression model using the enter method. A threshold of *p* < 0.05 was applied for inclusion in the final model. Based on the finalized independent predictors, a visualized nomogram was constructed using the “rms” package in R software, providing a quantitative tool for individualized risk prediction (24). Multicollinearity was assessed using the variance inflation factor (VIF); VIF < 10 was considered indicative of the absence of severe multicollinearity.

### Machine learning models

To further evaluate variable importance and provide cross-validation against the logistic regression model, Random Forest and extreme gradient boosting (XGBoost) machine learning models were additionally constructed. The Random Forest model quantified and ranked each variable’s contribution to classification using mean decrease in Gini impurity; hyperparameters (number of trees and maximum number of features) were determined via grid search with 5-fold cross-validation. Key hyperparameters for the XGBoost model (learning rate, maximum tree depth, and subsampling ratio) were similarly optimized through cross-validation (25).

### Model performance evaluation

Discrimination was assessed using the AUC. Pairwise AUC comparisons were performed using the DeLong method. Bootstrap resampling (1,000 iterations) was applied to calculate the optimism-corrected AUC for the nomogram and to evaluate its internal stability.

Calibration curves and the Hosmer-Lemeshow (H-L) goodness-of-fit test were used to evaluate the agreement between predicted probabilities and observed outcomes; H-L test *p* > 0.05 indicated satisfactory calibration. The Brier score was used to comprehensively assess prediction error, with lower values indicating greater predictive accuracy.

### Clinical utility

DCA was applied to estimate the net benefit of each model in guiding clinical decisions across a range of predefined threshold probabilities, thereby quantifying the relative clinical value of each model in practice.

### Assessment of incremental predictive value

In the validation cohort, NRI and IDI were used to quantitatively assess the incremental predictive value of the new model over conventional PSI and CURB-65. NRI quantifies the net improvement in correct upward reclassification of events and correct downward reclassification of non-events; IDI reflects the overall improvement in the separation of predicted probabilities between the new and reference models. Ninety-five percent confidence intervals (CIs) for both metrics were calculated using the bootstrap method (1,000 resamples); positive values with *p* < 0.05 indicate statistically significant incremental value. Scatter plots and risk reclassification bar charts were additionally constructed to visually demonstrate the direction and proportion of individual probability adjustments between the new and old models.

### Construction of the THM simplified integer score

To facilitate rapid bedside clinical assessment, a simplified integer THM score was derived from the three highest-weighted inflammatory-nutritional-metabolic composite indices in the multivariable logistic regression model (TyG index, MHR, and HALP index). Optimal binary cutoff values for each index were determined by the Youden index method (maximizing sensitivity + specificity − 1). Scoring weights were simplified and rounded in proportion to the relative regression coefficients, with each index assigned 2 points (TyG index ≥9.0: 2 points; MHR ≥ 0.52: 2 points; HALP index ≤20: 2 points), yielding a total score of 0–6. Patients were classified into low-risk (0–2 points), intermediate-risk (3–4 points), and high-risk (5–6 points) groups, and adverse outcome rates were reported for each stratum. The statistical significance of the risk stratification gradient was assessed using the Cochran-Armitage trend test. Discrimination comparison and incremental value assessment of the THM score relative to traditional scoring systems were performed using the same methods described above.

### Subgroup analyses

Pre-specified subgroup analyses were conducted according to age (<65 vs. ≥65 years), sex (male vs. female), diabetes history (yes vs. no), and PSI risk class (class I–III vs. IV–V) to evaluate the robustness and consistency of the core predictors across clinically distinct subpopulations. Heterogeneity of effect sizes across subgroups was assessed using interaction tests, with results presented as forest plots reporting odds ratios (ORs) and 95% CIs for each subgroup.

### Statistical analyses

Sample size was estimated *a priori* using the Hanley–McNeil method for comparing two correlated AUCs, assuming a target nomogram AUC of 0.85 versus a reference (PSI) AUC of 0.75, an expected event rate of 17%, a non-event-to-event ratio of approximately 5:1, two-sided *α* = 0.05, and power of 0.90; the required minimum sample size was 538 patients (≈91 events), which the present training cohort (*n* = 587, 103 events) and overall analytic cohort (*n* = 839, 148 events) comfortably exceed.

All statistical analyses were performed using R software (version 4.3.1), with the primary packages including “glmnet” (LASSO regression), “rms” (nomogram construction), “pROC” (ROC curves and DeLong test), “randomForest” (Random Forest), “xgboost” (XGBoost), “rmda” (DCA), and “ggplot2” (data visualization). Normally distributed continuous variables are expressed as mean ± standard deviation (SD); non-normally distributed variables are expressed as median (interquartile range [IQR]); categorical variables are expressed as frequency (proportion). Between-group comparisons were conducted using the independent-samples *t*-test for normally distributed continuous variables, the Mann–Whitney U test for non-normally distributed variables, and the chi-square test or Fisher’s exact test for categorical variables. All statistical tests were two-tailed, with *p* < 0.05 considered statistically significant.

## Results

### Baseline characteristics of the study population

A total of 839 hospitalized pneumonia patients were enrolled and randomly divided into a training cohort (*n* = 587) and a validation cohort (*n* = 252) at a 7:3 ratio. As shown in Table 1, no statistically significant differences were observed between the two cohorts in age, sex, BMI, comorbidities, clinical scores, or any of the inflammatory-nutritional-metabolic composite indices at baseline (all *p* > 0.05), indicating satisfactory between-group balance following random allocation. The overall incidence of short-term adverse outcomes (in-hospital ICU admission and/or IMV) was 17.6% (148/839); rates in the training and validation cohorts were 17.5% (103/587) and 17.9% (45/252), respectively, with no statistically significant difference (*p* = 0.899). Within the composite endpoint (*n* = 148), 90 patients (60.8%) had isolated ICU admission without IMV, while 58 patients (39.2%) received IMV with concurrent ICU admission; no patient received IMV without ICU admission. A pre-specified sensitivity analysis using IMV alone as the outcome yielded a nomogram AUC of 0.857 (95% CI: 0.811–0.903) in the validation cohort, closely paralleling the primary composite analysis (AUC = 0.848) and supporting that the model’s discriminative capacity is anchored in respiratory-failure physiology rather than in non-respiratory drivers of ICU triage.

**Table 1:** Comparison of baseline characteristics between training and validation sets.

Variable	Overall (*n* = 839)	Training set (*n* = 587)	Validation set (*n* = 252)	*P* value
Age (years)	64.2 ± 16.0	64.3 ± 15.8	63.9 ± 16.4	0.723
Male, *n* (%)	524 (62.5%)	364 (62.0%)	160 (63.5%)	0.672
BMI (kg/m^2^)	22.7 ± 3.8	22.8 ± 3.8	22.6 ± 3.9	0.481
Smoking history, *n* (%)	280 (33.4%)	196 (33.4%)	84 (33.3%)	0.984
Long-term alcohol use, *n* (%)	126 (15.0%)	88 (15.0%)	38 (15.1%)	0.970
COPD, *n* (%)	182 (21.7%)	130 (22.1%)	52 (20.6%)	0.612
Diabetes mellitus, *n* (%)	222 (26.5%)	156 (26.6%)	66 (26.2%)	0.894
Heart failure, *n* (%)	95 (11.3%)	68 (11.6%)	27 (10.7%)	0.706
Coronary artery disease, *n* (%)	161 (19.2%)	114 (19.4%)	47 (18.7%)	0.796
Chronic kidney disease, *n* (%)	86 (10.3%)	60 (10.2%)	26 (10.3%)	0.963
Malignancy, *n* (%)	120 (14.3%)	84 (14.3%)	36 (14.3%)	0.998
Cerebrovascular disease, *n* (%)	92 (11.0%)	65 (11.1%)	27 (10.7%)	0.868
Short-term adverse outcome, *n* (%)	148 (17.6%)	103 (17.5%)	45 (17.9%)	0.899
CURB-65 score, M (IQR)	2 (1–3)	2 (1–3)	2 (1–3)	0.841
PSI class III–V, *n* (%)	418 (49.8%)	289 (49.2%)	129 (51.2%)	0.581
NLR	8.22 (4.44–14.90)	8.14 (4.36–14.82)	8.32 (4.58–15.04)	0.764
HALP index	28.26 (14.64–43.96)	28.46 (14.82–44.12)	27.94 (14.36–43.68)	0.826
TyG index	8.91 ± 0.84	8.92 ± 0.83	8.88 ± 0.86	0.537
MHR	0.48 (0.29–0.73)	0.48 (0.29–0.73)	0.47 (0.30–0.72)	0.912

Normally distributed continuous variables are presented as mean ± SD; skewed variables as M (IQR); categorical variables as *n* (%). *P* values reflect comparisons between training and validation sets; no statistically significant differences were observed, indicating adequate balance. NLR, neutrophil-to-lymphocyte ratio; HALP = hemoglobin × albumin × lymphocyte/platelet; TyG = ln[triglycerides (mg/dL) × fasting glucose (mg/dL)/2]; MHR, monocyte-to-HDL-cholesterol ratio.

Within the training cohort, patients who experienced adverse outcomes were older (68.8 ± 14.9 vs. 62.9 ± 15.7 years, *p* = 0.001), had lower BMI (22.1 ± 3.8 vs. 23.0 ± 3.8 kg/m^2^, *p* = 0.038), and had significantly higher rates of COPD (33.0% vs. 19.8%, *p* = 0.004), diabetes mellitus (36.9% vs. 24.4%, *p* = 0.010), and heart failure (19.4% vs. 9.9%, *p* = 0.009) compared with patients with good prognosis (Table 2). Regarding composite inflammatory-nutritional-metabolic indices, NLR (14.82 vs. 6.44, *p* < 0.001), MHR (0.68 vs. 0.41, *p* < 0.001), and TyG index (9.27 ± 0.86 vs. 8.81 ± 0.78, *p* < 0.001) were all significantly elevated, while the HALP index (14.28 vs. 32.64, *p* < 0.001) was markedly reduced in the adverse outcome group. Secondary outcome analyses (Supplementary Table 3) demonstrated that patients with adverse outcomes had significantly prolonged hospital stays (15.2 vs. 9.1 days, *p* < 0.001) and substantially higher rates of in-hospital complications, including sepsis (59.5% vs. 12.0%), septic shock (29.1% vs. 2.6%), ARDS (46.6% vs. 3.3%), and AKI (37.2% vs. 9.0%) (all *p* < 0.001).

**Table 2:** Comparison of baseline and laboratory variables between adverse outcome and good outcome groups in the training set.

Variable	Adverse outcome (*n* = 103)	Good outcome (*n* = 484)	*P* value
General characteristics			
Age (years)	68.8 ± 14.9	62.9 ± 15.7	0.001
Male, *n* (%)	67 (65.0%)	297 (61.4%)	0.503
BMI (kg/m^2^)	22.1 ± 3.8	23.0 ± 3.8	0.038
Smoking history, *n* (%)	44 (42.7%)	152 (31.4%)	0.027
Long-term alcohol use, *n* (%)	19 (18.4%)	69 (14.3%)	0.283
Comorbidities			
COPD, *n* (%)	34 (33.0%)	96 (19.8%)	0.004
Diabetes mellitus, *n* (%)	38 (36.9%)	118 (24.4%)	0.010
Heart failure, *n* (%)	20 (19.4%)	48 (9.9%)	0.009
Coronary artery disease, *n* (%)	27 (26.2%)	87 (18.0%)	0.061
Chronic kidney disease, *n* (%)	17 (16.5%)	43 (8.9%)	0.024
Malignancy, *n* (%)	22 (21.4%)	62 (12.8%)	0.031
Cerebrovascular disease, *n* (%)	16 (15.5%)	49 (10.1%)	0.127
Bronchial asthma, *n* (%)	10 (9.7%)	38 (7.9%)	0.551
Liver cirrhosis, *n* (%)	6 (5.8%)	15 (3.1%)	0.193
Admission vital signs			
Heart rate (beats/min)	103.2 ± 19.4	89.8 ± 17.6	<0.001
Respiratory rate (breaths/min)	24.8 ± 6.2	20.4 ± 4.8	<0.001
Systolic BP (mmHg)	125.2 ± 22.8	134.8 ± 20.9	<0.001
Diastolic BP (mmHg)	74.4 ± 15.4	79.2 ± 13.6	0.003
Body temperature (°C)	38.6 ± 0.9	38.2 ± 0.8	<0.001
SpO₂ (%)	90.3 ± 7.6	94.6 ± 5.3	<0.001
Altered consciousness, *n* (%)	24 (23.3%)	37 (7.6%)	<0.001
Clinical scores			
PSI class III–V, *n* (%)	84 (81.6%)	205 (42.4%)	<0.001
CURB-65 score, M (IQR)	3 (2–4)	1 (1–2)	<0.001
CURB-65 ≥ 2, *n* (%)	72 (69.9%)	152 (31.4%)	<0.001
Treatment			
High-flow nasal cannula, *n* (%)	44 (42.7%)	70 (14.5%)	<0.001
Non-invasive ventilation, *n* (%)	34 (33.0%)	30 (6.2%)	<0.001
Systemic corticosteroids, *n* (%)	41 (39.8%)	111 (22.9%)	0.001
Combination antibiotics, *n* (%)	60 (58.3%)	186 (38.4%)	<0.001
Complete blood count			
WBC (×10^9^/L)	12.6 (9.1–17.4)	9.5 (7.2–12.7)	<0.001
Neutrophil count (×10^9^/L)	10.6 (7.3–15.1)	7.3 (5.3–10.0)	<0.001
Lymphocyte count (×10^9^/L)	0.68 (0.46–0.97)	1.12 (0.79–1.57)	<0.001
Monocyte count (×10^9^/L)	0.58 (0.39–0.82)	0.48 (0.32–0.68)	0.006
Eosinophil count (×10^9^/L)	0.02 (0.00–0.07)	0.08 (0.02–0.17)	<0.001
Platelet count (×10^9^/L)	198 (142–264)	222 (166–294)	0.008
Hemoglobin (g/L)	108.2 ± 24.8	122.9 ± 22.4	<0.001
Inflammatory markers			
C-reactive protein (mg/L)	142.6 (82.4–218.8)	67.4 (32.2–126.8)	<0.001
Procalcitonin (ng/mL)	2.84 (0.68–12.96)	0.37 (0.08–1.42)	<0.001
Interleukin-6 (pg/mL)	184.2 (62.8–486.4)	47.6 (17.8–141.2)	<0.001
Ferritin (μg/L)	674.8 (318.6–1228.4)	264.8 (122.6–562.4)	<0.001
Nutritional markers			
Albumin (g/L)	28.6 ± 6.2	34.8 ± 6.5	<0.001
Prealbumin (mg/L)	98.4 (63.6–146.2)	168.8 (125.6–218.4)	<0.001
Transferrin (g/L)	1.64 (1.16–2.10)	2.22 (1.76–2.66)	<0.001
Metabolic and lipid parameters			
Fasting glucose (mmol/L)	8.62 (6.44–12.88)	6.78 (5.58–9.22)	<0.001
Triglycerides (mmol/L)	1.46 (0.96–2.18)	1.26 (0.86–1.84)	0.048
HDL-cholesterol (mmol/L)	0.82 ± 0.26	1.08 ± 0.32	<0.001
LDL-cholesterol (mmol/L)	2.24 ± 0.86	2.46 ± 0.93	0.028
Hepatorenal function and coagulation			
Creatinine (μmol/L)	102.8 (72.6–168.4)	77.2 (58.8–107.6)	<0.001
Blood urea nitrogen (mmol/L)	11.2 (7.4–18.4)	7.3 (4.9–11.4)	<0.001
D-dimer (mg/L)	3.88 (1.62–8.24)	1.20 (0.54–2.78)	<0.001
Fibrinogen (g/L)	4.82 (3.68–6.22)	4.10 (3.22–5.40)	0.002
Cardiac biomarkers and blood gas			
NT-proBNP (pg/mL)	1,298 (484–4,268)	278 (82–834)	<0.001
Cardiac troponin I (ng/mL)	0.082 (0.017–0.348)	0.015 (0.006–0.059)	<0.001
PaO₂/FiO₂ (mmHg)	168.4 ± 82.6	271.2 ± 92.4	<0.001
Inflammatory-nutritional-metabolic composite indices			
NLR	14.82 (9.24–22.68)	6.44 (3.86–10.72)	<0.001
PLR	298.6 (184.2–428.4)	180.8 (116.6–264.2)	<0.001
LMR	1.18 (0.72–1.92)	2.28 (1.50–3.44)	<0.001
ELR	0.03 (0.00–0.09)	0.07 (0.02–0.15)	<0.001
HALP index	14.28 (8.52–22.84)	32.64 (20.42–47.96)	<0.001
TyG index	9.27 ± 0.86	8.81 ± 0.78	<0.001
MHR	0.68 (0.42–1.02)	0.41 (0.27–0.63)	<0.001

Skewed continuous variables are presented as M (IQR); normally distributed variables as mean ± SD; categorical variables as *n* (%). Independent *t*-test or Mann–Whitney U test was used for continuous variables; χ^2^ test or Fisher’s exact test for categorical variables. Short-term adverse outcome was defined as in-hospital ICU admission and/or invasive mechanical ventilation. NLR, neutrophil-to-lymphocyte ratio; PLR, platelet-to-lymphocyte ratio; LMR, lymphocyte-to-monocyte ratio; ELR, eosinophil-to-lymphocyte ratio; HALP = hemoglobin × albumin × lymphocyte/platelet; TyG = ln[triglycerides (mg/dL) × fasting glucose (mg/dL)/2]; MHR, monocyte-to-HDL-cholesterol ratio.

### Correlation analysis of core indices and predictor selection

**Figure 1:**
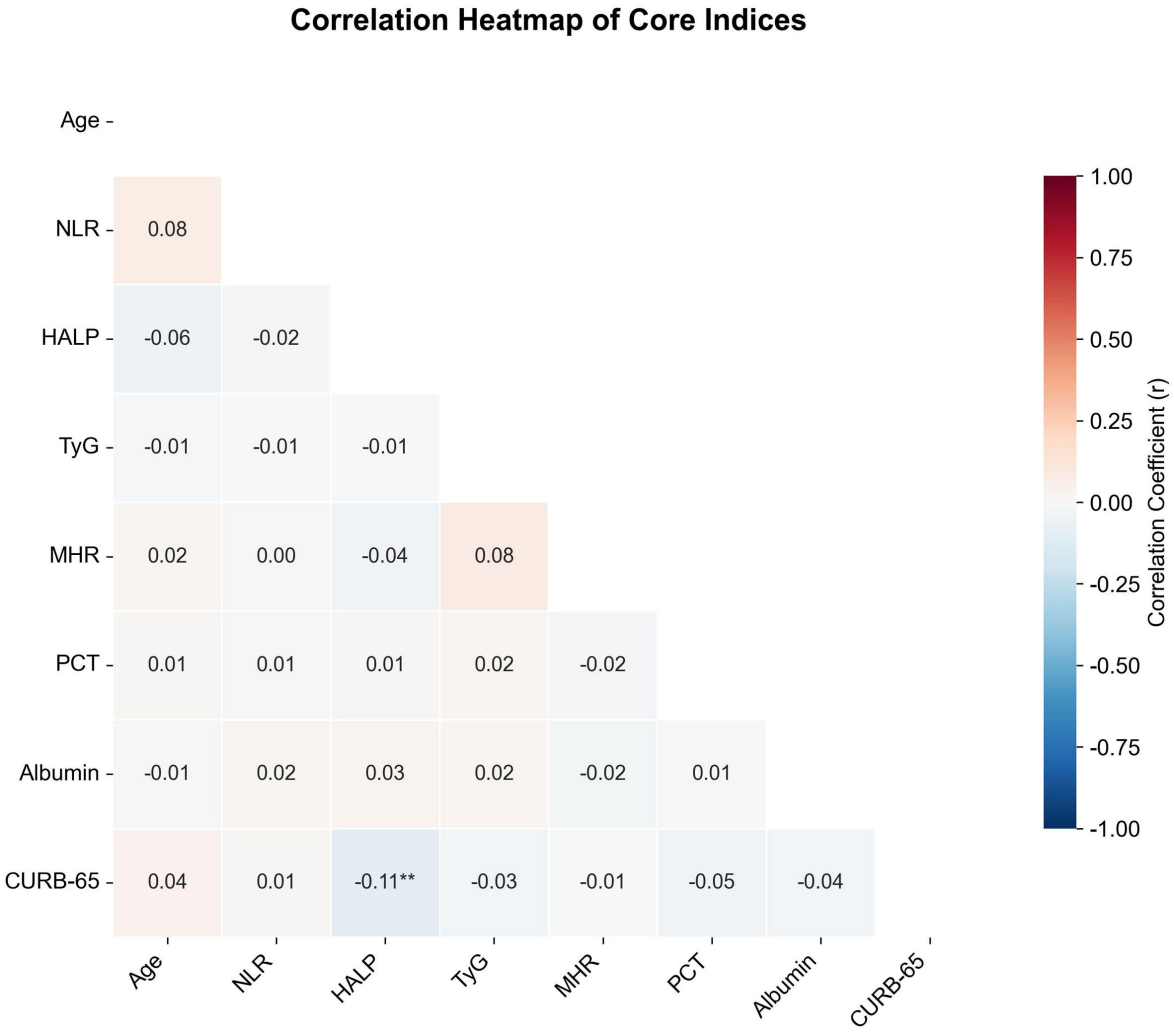
Correlation heatmap of core clinical features and composite indices. Values in the matrix represent pairwise Pearson correlation coefficients (*r*) between variables. The color scale on the right indicates the direction and magnitude of correlations: red hues represent positive correlations, and blue hues represent negative correlations, with deeper colors indicating stronger associations. Results demonstrate that the constructed inflammatory, nutritional, and metabolic indices maintain relative independence, confirming that their joint use does not introduce severe multicollinearity. Abbreviations: NLR, neutrophil-to-lymphocyte ratio; HALP, hemoglobin × albumin × lymphocyte/platelet index; TyG, triglyceride-glucose index; MHR, monocyte-to-high-density lipoprotein cholesterol ratio; PCT, procalcitonin; CURB-65, pneumonia severity score.

To assess multicollinearity among the candidate inflammatory, nutritional, and metabolic indices, a correlation matrix analysis of core variables was first performed in the training cohort. As shown in Figure 1, Pearson correlation analysis revealed that the absolute values of pairwise correlation coefficients (r) among all indices were uniformly low. The only statistically significant correlation was a weak negative association between the HALP index and CURB-65 score (r = −0.11, *p* < 0.01); all remaining pairwise correlation coefficients had absolute values not exceeding 0.08, confirming that the composite indices maintained satisfactory mutual independence and that their joint use would not introduce severe multicollinearity.

**Figure 2:**
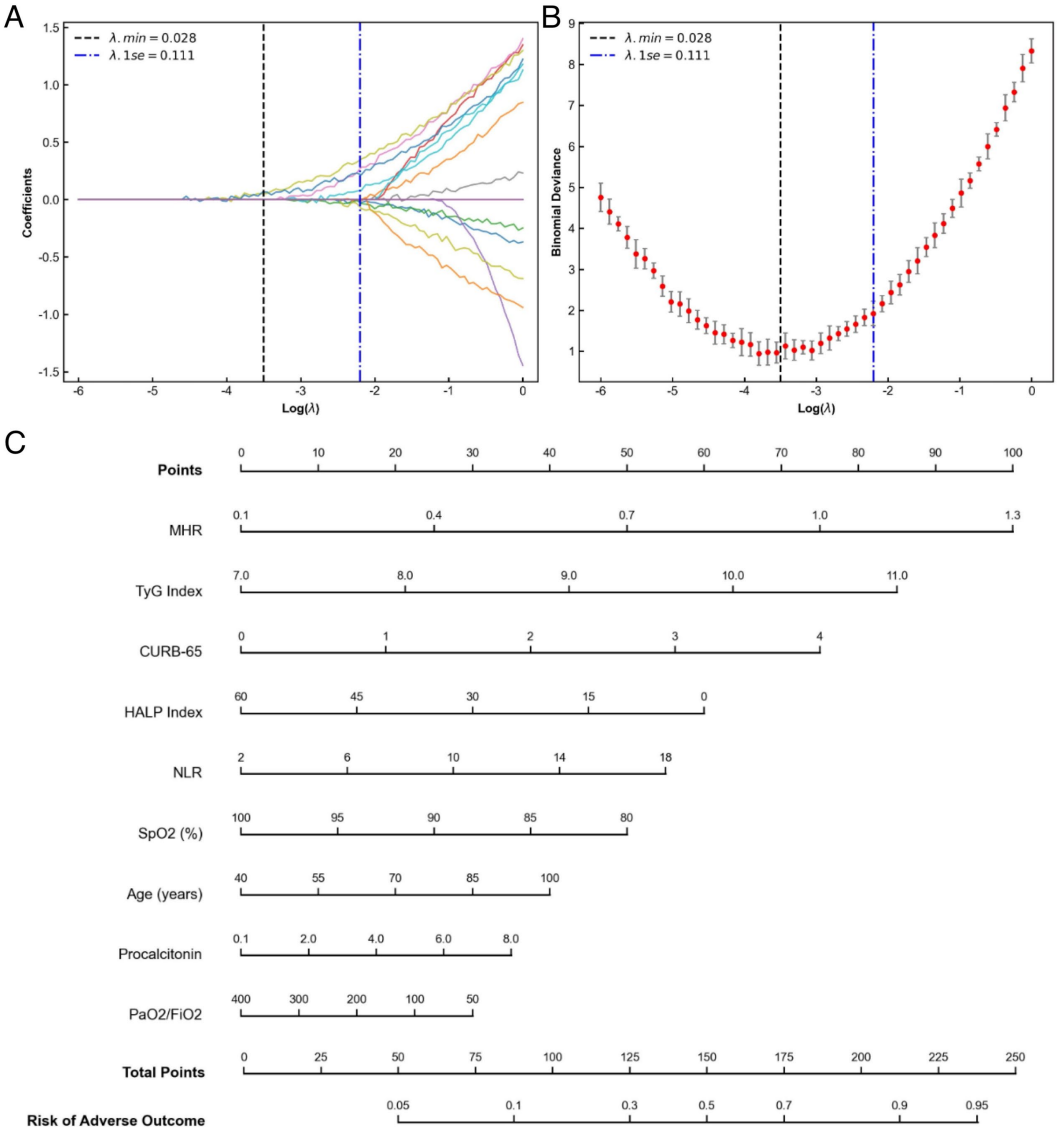
Variable selection and nomogram construction for the prediction model. **(A)** LASSO regression coefficient profile plot. The *x*-axis represents the log-transformed penalty parameter log(*λ*), and the *y*-axis represents the standardized regression coefficients of candidate variables. As λ increases, coefficients progressively shrink toward zero. **(B)** Ten-fold cross-validation error plot. Vertical dashed lines indicate the penalty parameter corresponding to the minimum cross-validation error (λ.min = 0.028) and the optimal coefficient within one standard error (λ.1se). The value of λ.min was selected as the threshold, retaining 11 candidate predictors with non-zero coefficients. **(C)** Nomogram for predicting short-term adverse outcomes, constructed from the nine independent predictors finalized in multivariable logistic regression. For each predictor, the patient’s value is located on the corresponding axis and projected vertically upward to the “Points” axis to obtain the individual item score; the sum of all nine item scores yields the “Total Points,” which is then projected downward to the “Risk of Adverse Outcome” axis at the bottom to obtain the individualized predicted probability of short-term adverse outcome. Positive predictors (e.g., MHR and TyG index, indicating increased risk) are scaled in ascending order from left to right; negative protective factors (e.g., SpO_2_ and HALP index, indicating reduced risk) are scaled in descending order from right to left.

Twenty-five candidate clinical and laboratory variables were then entered into LASSO regression (Supplementary Table 1; Figures 2A,B). Ten-fold cross-validation identified an optimal penalty parameter of *λ*.min = 0.028, retaining 11 variables with non-zero coefficients: age, SpO₂, NLR, HALP index, TyG index, MHR, PCT, PaO₂/FiO₂, CURB-65 score, COPD, and diabetes mellitus. These 11 variables were entered into multivariable logistic regression (Table 3), whereupon COPD (*p* = 0.069) and diabetes mellitus (*p* = 0.113) failed to reach statistical significance and were excluded. The final model contained nine independent predictors: MHR (OR = 2.063, 95% CI: 1.362–3.124, *p* = 0.001), TyG index (OR = 1.619, 95% CI: 1.192–2.201, *p* = 0.002), NLR (OR = 1.060, 95% CI: 1.028–1.092, *p* < 0.001), HALP index (OR = 0.965, 95% CI: 0.948–0.981, *p* < 0.001), SpO₂ (OR = 0.920, 95% CI: 0.894–0.946, *p* < 0.001), PaO₂/FiO₂ (OR = 0.995, 95% CI: 0.992–0.998, *p* = 0.003), CURB-65 score (OR = 1.422, 95% CI: 1.108–1.824, *p* = 0.006), PCT (OR = 1.039, 95% CI: 1.014–1.064, *p* = 0.002), and age (OR = 1.019, 95% CI: 1.004–1.035, *p* = 0.014). Multicollinearity assessment confirmed VIF values below 3.0 for all variables (range: 1.12–2.86), indicating stable and reliable regression coefficient estimation.

**Table 3:** Multivariable logistic regression analysis (based on LASSO-selected variables, training set, *n* = 587).

Variable	*β* coefficient	OR	95% CI	*P* value
Age (years)	0.019	1.019	1.004–1.035	0.014
SpO₂ (%)	−0.084	0.920	0.894–0.946	<0.001
NLR	0.058	1.060	1.028–1.092	<0.001
HALP index	−0.036	0.965	0.948–0.981	<0.001
TyG index	0.482	1.619	1.192–2.201	0.002
MHR	0.724	2.063	1.362–3.124	0.001
Procalcitonin (ng/mL)	0.038	1.039	1.014–1.064	0.002
PaO₂/FiO₂ (mmHg)	−0.005	0.995	0.992–0.998	0.003
CURB-65 score	0.352	1.422	1.108–1.824	0.006

All 11 LASSO-selected variables were entered simultaneously using the Enter method; COPD (*P* = 0.069) and diabetes mellitus (*P* = 0.113) did not reach statistical significance and were excluded; the final model comprised 9 variables used to construct the nomogram. VIF values for all retained variables were <3.0 (range: 1.12–2.86), indicating no substantial multicollinearity. Hosmer-Lemeshow test *p* = 0.608.

### Nomogram construction

A visualized nomogram was constructed based on the nine independent predictors (Figure 2C). For each predictor, the corresponding value on its respective axis is identified and projected vertically upward to the “Points” axis to obtain its individual score; the sum of all nine scores yields the “Total Points” (range: 0–250), which is then projected downward onto the “Risk of Adverse Outcome” axis at the bottom to read the individualized predicted probability of short-term adverse outcome (range: 0.05–0.95). MHR and TyG index occupied wider scoring ranges on the nomogram, reflecting their greater contribution to the total score, consistent with the multivariable regression results.

### Random Forest variable importance analysis

**Figure 3:**
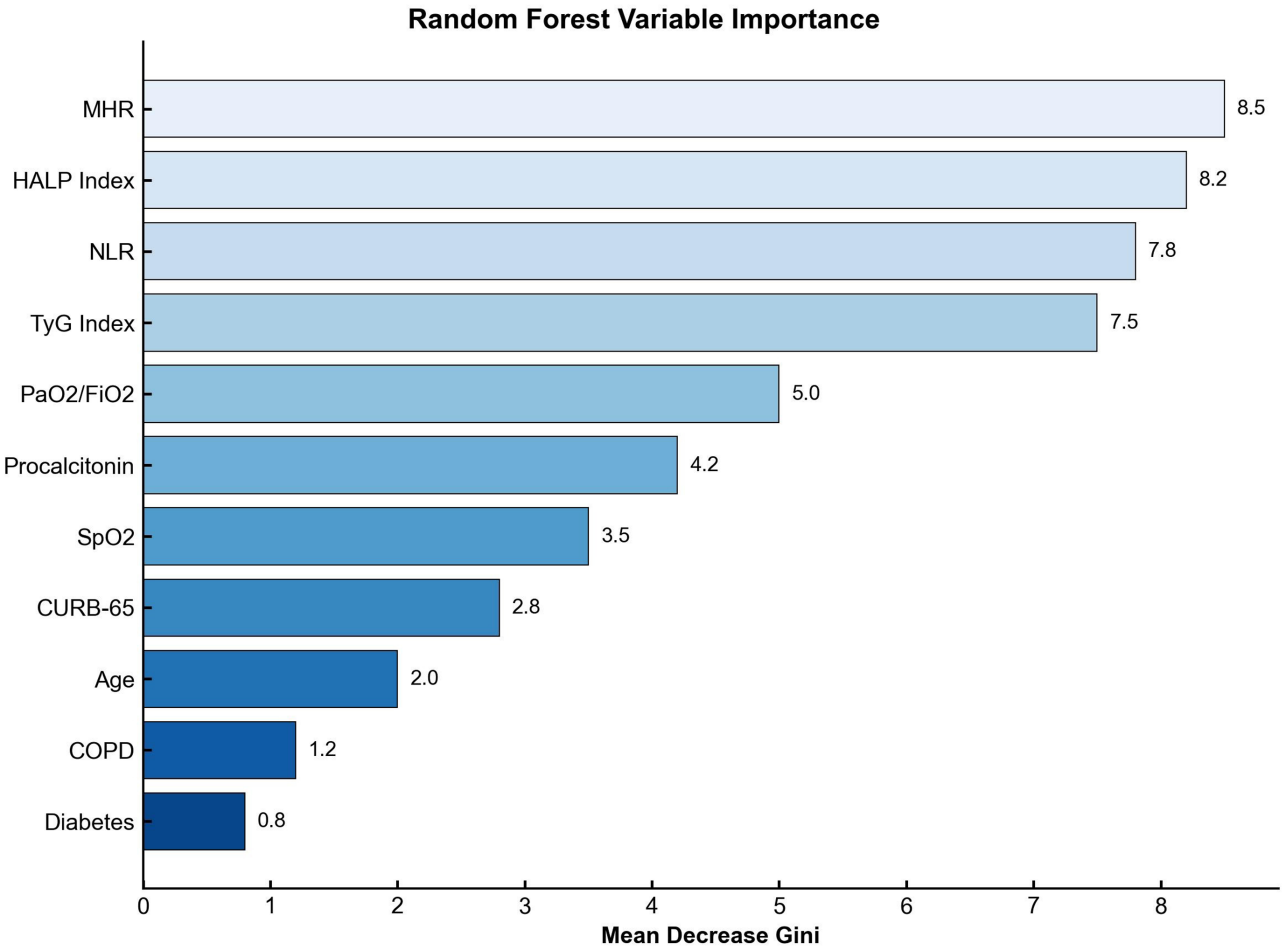
Random forest variable importance ranking (training cohort). The *x*-axis represents the mean decrease in Gini impurity; larger values indicate greater contribution to node splitting within classification trees and thus higher global importance for predicting short-term adverse outcomes. Composite inflammatory-nutritional-metabolic indices including MHR, HALP index, NLR, and TyG index ranked highest, demonstrating outstanding predictive value.

The Random Forest algorithm was used to independently validate and quantitatively rank the global importance of candidate variables (Figure 3). As measured by mean decrease in Gini impurity, MHR (8.5), HALP index (8.2), NLR (7.8), and TyG index (7.5) ranked highest among all variables, followed by PaO₂/FiO₂ (5.0), PCT (4.2), SpO₂ (3.5), and CURB-65 (2.8), with age (2.0), COPD (1.2), and diabetes mellitus (0.8) exhibiting relatively lower importance. These results were highly concordant with the key predictors identified in multivariable logistic regression, further confirming the central role of inflammatory-nutritional-metabolic composite indices in predicting short-term adverse outcomes.

### Model predictive performance evaluation

**Figure 4:**
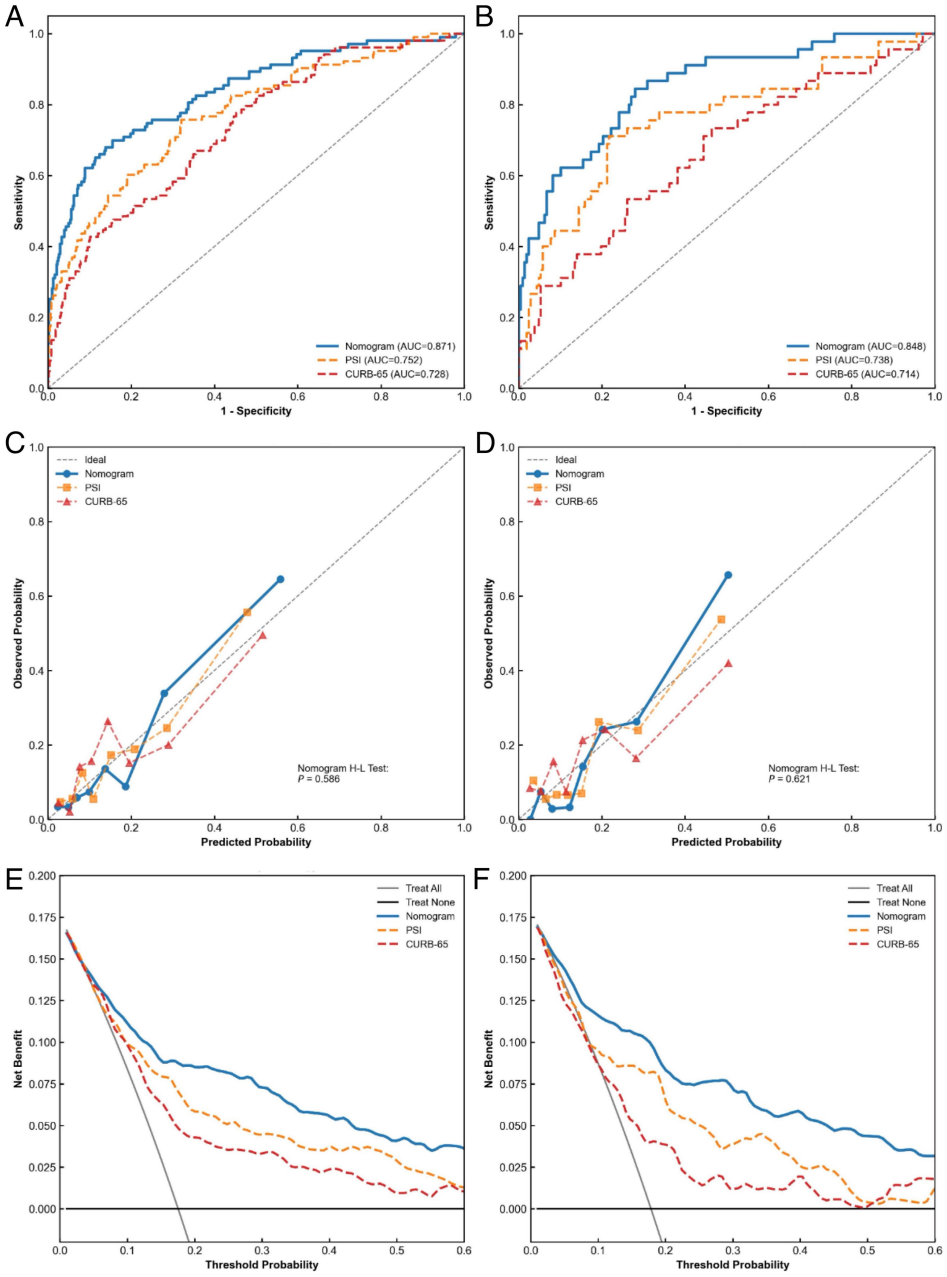
Comprehensive validation of predictive performance and clinical utility of the nomogram versus traditional scoring systems. **(A,B)** ROC curves for the training and validation cohorts, respectively, comparing the discrimination (AUC) of the nomogram, PSI, and CURB-65. **(C,D)** Calibration curves for the training and validation cohorts, respectively, with the H-L test assessing agreement between predicted probabilities and observed event rates (the 45° dashed line represents perfect calibration). **(E,F)** DCA for the training and validation cohorts, respectively, demonstrating net benefit of each model in guiding clinical intervention across a range of threshold probabilities. The nomogram is superior to traditional scores in terms of discrimination, calibration, and clinical benefit.

Detailed performance comparisons across all prediction models in the training and validation cohorts are presented in Table 4. ROC curve analysis (Figures 4A,B) showed that in the training cohort, the nomogram achieved an AUC of 0.871 (95% CI: 0.837–0.905), significantly outperforming PSI (AUC = 0.752) and CURB-65 (AUC = 0.728); in the validation cohort, the nomogram AUC remained 0.848 (95% CI: 0.802–0.894), still significantly superior to PSI (AUC = 0.738, *p* = 0.002) and CURB-65 (AUC = 0.714, *p* < 0.001). After 1,000 bootstrap resampling iterations, the optimism-corrected AUC of the nomogram was 0.838, with an optimism correction of only 0.010, indicating no substantial overfitting. Random Forest (validation cohort AUC = 0.868) and XGBoost (validation cohort AUC = 0.862) showed comparable discrimination to the nomogram, with no statistically significant differences among the three (all *p* > 0.05).

**Table 4:** Predictive performance of all models in training and validation sets.

Model	Dataset	AUC (95% CI)	Sensitivity (%)	Specificity (%)	PPV (%)	NPV (%)	Brier score
Nomogram (9 variables)	Training	0.871 (0.837–0.905)	79.6	84.5	57.4	93.8	0.098
	Validation	0.848 (0.802–0.894)	77.1	82.1	54.8	92.5	0.106
	Bootstrap-corrected	0.838 (0.801–0.875)	–	–	–	–	–
Random Forest	Training	0.896 (0.866–0.926)	83.5	86.8	62.4	95.2	0.088
	Validation	0.868 (0.826–0.910)	80.0	84.1	57.1	93.7	0.097
XGBoost	Training	0.889 (0.858–0.920)	82.5	85.7	60.9	94.7	0.091
	Validation	0.862 (0.819–0.905)	79.1	83.4	56.3	93.3	0.100
PSI score	Training	0.752 (0.707–0.797)	73.8	72.3	41.6	91.2	0.136
	Validation	0.738 (0.681–0.795)	71.1	70.8	38.1	90.0	0.143
CURB-65	Training	0.728 (0.681–0.775)	71.8	71.9	40.8	90.7	0.141
	Validation	0.714 (0.655–0.773)	68.9	70.2	37.0	89.5	0.147

DeLong test: Nomogram vs PSI, *P* = 0.002; Nomogram vs CURB-65, *P* < 0.001 (validation set). AUC differences among the three machine learning models were not statistically significant (all *P* > 0.05). Bootstrap resampling: 1,000 iterations; optimism correction = 0.010. AUC, area under the receiver operating characteristic curve; PPV, positive predictive value; NPV, negative predictive value; lower Brier score indicates greater predictive accuracy.

Calibration curves (Figures 4C,D) and H-L test results showed *p* = 0.586 in the training cohort and *p* = 0.621 in the validation cohort, confirming good agreement between predicted probabilities and observed event rates and satisfactory calibration. DCA (Figures 4E,F) showed that across a threshold probability range of 0–0.6, the net benefit curve of the nomogram consistently surpassed those of PSI, CURB-65, and the “treat all” reference line, confirming superior clinical utility across a broad range of decision thresholds.

### Subgroup analyses

**Figure 5:**
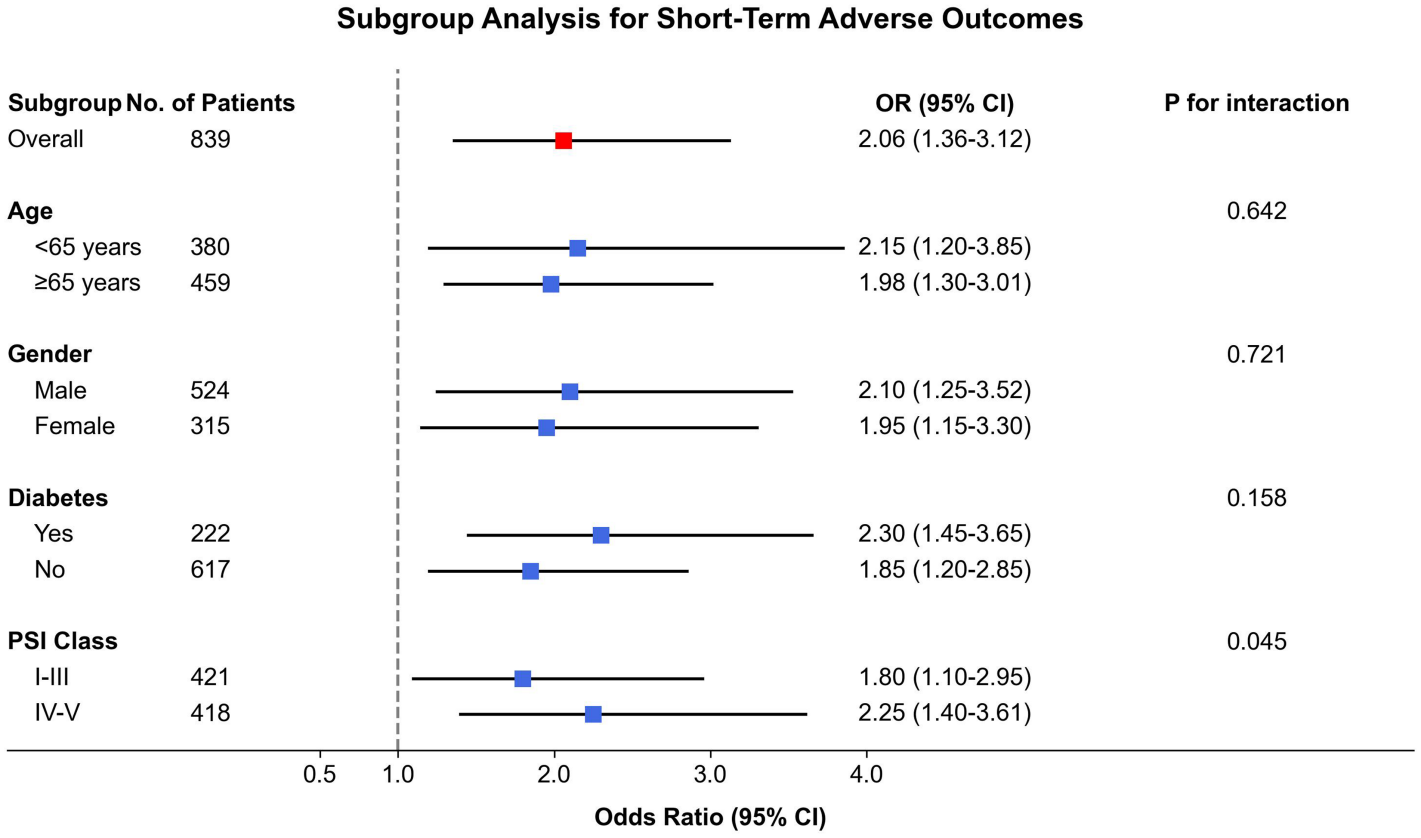
Forest plot of subgroup analyses for predicting short-term adverse outcomes. Robustness of the core predictors was evaluated across subgroups defined by age, sex, diabetes history, and PSI risk class. Squares represent ORs and horizontal lines represent 95% CIs. The red square represents the overall effect in the study population; blue squares represent stratified effects in each subgroup. The vertical gray dashed line is the null-hypothesis reference line (OR = 1.0). *p* values for interaction confirm that the predictive index maintained stable and significant predictive performance across all heterogeneous subpopulations examined.

Pre-specified subgroup analyses were conducted to evaluate the robustness of the core predictors’ performance according to age, sex, diabetes history, and PSI risk class (Figure 5). The overall OR for the entire cohort was 2.06 (95% CI: 1.36–3.12; *n* = 839). No significant heterogeneity in effect size was observed across age (P for interaction = 0.642), sex (P for interaction = 0.721), or diabetes (P for interaction = 0.158) subgroups, indicating good cross-population generalizability. In the PSI risk-class subgroup analysis, the OR was 2.25 (95% CI: 1.40–3.61) among high-risk patients (PSI class IV–V; *n* = 418), marginally higher than 1.80 (95% CI: 1.10–2.95) among low-to-moderate-risk patients (PSI class I–III; *n* = 421), with a statistically significant interaction (P for interaction = 0.045), suggesting that the model may achieve even finer risk discrimination among patients with more severe illness.

### Incremental predictive value of the new model over traditional scores

**Figure 6:**
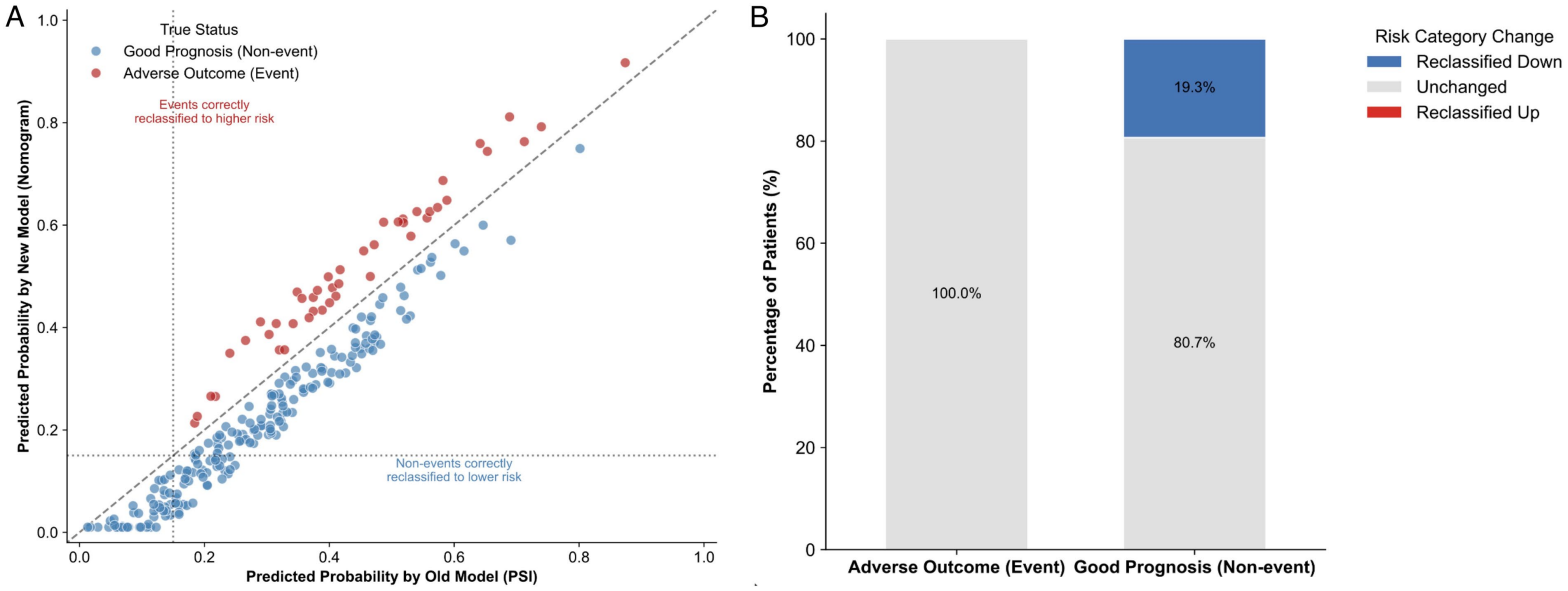
Visualization of net reclassification improvement of the new model (nomogram) versus the conventional model (PSI) for short-term adverse outcomes. **(A)** Scatter plot of predicted probability changes at the individual patient level. The *x*- and *y*-axes represent the predicted risk probabilities by the old and new models, respectively. Red and blue dots represent patients who experienced adverse outcomes (Event) and good prognosis (Non-event), respectively. The figure illustrates that the new model correctly upwardly reclassified many actual adverse-outcome patients (red dots above the diagonal) and correctly excluded risk for good-prognosis patients (blue dots below the diagonal). **(B)** Risk reclassification percentage bar chart stratified by actual outcome, quantifying the proportion of patients whose risk tier was correctly upgraded (red) or correctly downgraded (blue) by the new model at the predefined threshold, confirming its significant incremental clinical value in precise risk stratification.

NRI and IDI analyses (Table 5) demonstrated that in the validation cohort, the nomogram achieved an NRI of 0.278 (95% CI: 0.158–0.398, *p* < 0.001) and IDI of 0.121 (95% CI: 0.084–0.158, *p* < 0.001) relative to PSI, and an NRI of 0.306 (95% CI: 0.184–0.428, *p* < 0.001) and IDI of 0.134 (95% CI: 0.096–0.172, *p* < 0.001) relative to CURB-65. Risk reclassification visualization (Figure 6) intuitively illustrated the incremental clinical value of the new model: while PSI had already classified all patients with adverse outcomes as high-risk, the nomogram simultaneously maintained 100% high-risk capture (zero missed cases) and successfully downgraded 19.3% of good-prognosis patients who had been falsely overclassified by PSI to low-risk (Figure 6B), effectively reducing unnecessary escalation of care and waste of medical resources without any sacrifice in sensitivity.

**Table 5:** Incremental predictive value of the new model over PSI and CURB-65 scores (NRI/IDI analysis, validation set).

Comparison	NRI (95% CI)	NRI *P* value	IDI (95% CI)	IDI *P* value
Nomogram vs. PSI	0.278 (0.158–0.398)	<0.001	0.121 (0.084–0.158)	<0.001
Nomogram vs. CURB-65	0.306 (0.184–0.428)	<0.001	0.134 (0.096–0.172)	<0.001
THM score vs. PSI	0.214 (0.096–0.332)	<0.001	0.084 (0.054–0.114)	<0.001
THM score vs. CURB-65	0.242 (0.124–0.360)	<0.001	0.096 (0.064–0.128)	<0.001

NRI, net reclassification improvement; IDI, integrated discrimination improvement. 95% CIs were derived from 1,000 bootstrap resamples. Positive values indicate superior reclassification over the comparator. Analysis was performed in the internal validation set (*n* = 252).

### Construction and validation of the THM simplified integer score

**Figure 7:**
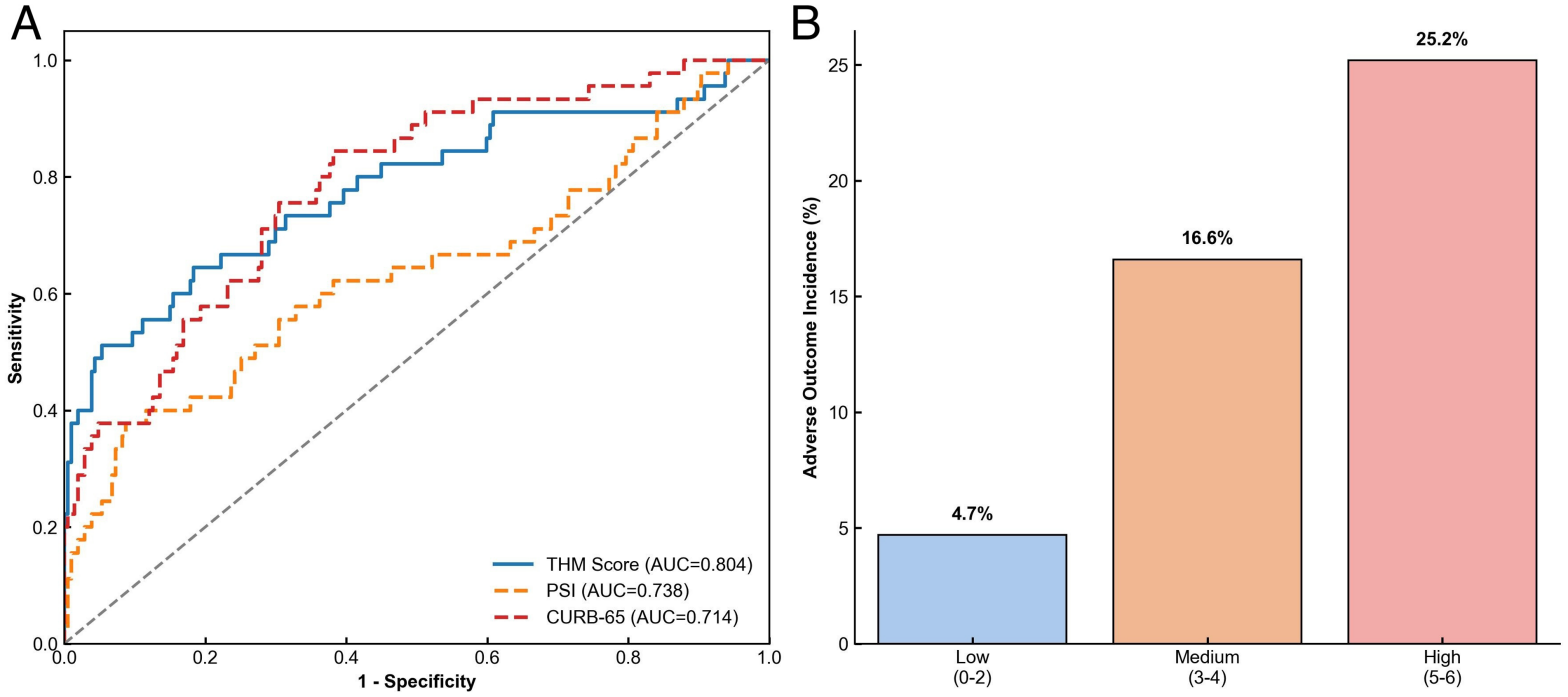
Construction, validation, and risk stratification performance of the THM composite score. **(A)** ROC curve comparison of the THM score (integrating TyG index, MHR, and HALP index), PSI, and CURB-65 for predicting short-term adverse outcomes, demonstrating superior independent discrimination for the THM score (AUC = 0.804). **(B)** Risk stratification bar chart based on the THM score, classifying patients into low-risk (0–2 points), intermediate-risk (3–4 points), and high-risk (5–6 points) groups. Adverse outcome rates of 4.7, 16.6, and 25.2% across groups show a statistically significant gradient trend, confirming the substantial clinical utility of this simplified integer score for rapid assessment in the emergency department or at the bedside.

To enhance the feasibility of rapid bedside clinical assessment, the THM simplified integer score was constructed from the three highest-weighted inflammatory-nutritional-metabolic composite indices in the multivariable logistic regression model (Supplementary Table 2). Optimal cutoff values were determined using the Youden index method in conjunction with the relative regression coefficients (TyG index *β* = 0.482, MHR *β* = 0.724, HALP index |*β*| = 0.036). Each index was assigned 2 points (TyG index ≥9.0: 2 points; MHR ≥ 0.52: 2 points; HALP index ≤20: 2 points), yielding a total score of 0–6. ROC curve analysis (Figure 7A) showed that the THM score achieved an AUC of 0.804 (95% CI: 0.763–0.845), superior to PSI (AUC = 0.738) and CURB-65 (AUC = 0.714). Risk stratification analysis (Figure 7B) demonstrated short-term adverse outcome rates of 4.7, 16.6, and 25.2% in the low-risk (0–2 points; *n* = 148), intermediate-risk (3–4 points; *n* = 386), and high-risk (5–6 points; *n* = 305) groups, respectively, with a statistically significant gradient trend (Cochran-Armitage trend test *p* < 0.001). Using the low-risk group as reference, the OR was 3.96 (95% CI: 1.74–9.02, *p* = 0.001) for the intermediate-risk group and 6.72 (95% CI: 2.96–15.28, *p* < 0.001) for the high-risk group. NRI/IDI analyses further confirmed significant incremental predictive value of the THM score over both PSI (NRI = 0.214, *p* < 0.001; IDI = 0.084, *p* < 0.001) and CURB-65 (NRI = 0.242, *p* < 0.001; IDI = 0.096, *p* < 0.001).

## Discussion

This study developed and validated a nomogram integrating multidimensional inflammatory-nutritional-metabolic composite indices for the early identification of short-term adverse outcomes in hospitalized pneumonia patients. The model incorporated nine independent predictors—MHR, TyG index, NLR, HALP index, SpO₂, PaO₂/FiO₂, CURB-65 score, PCT, and age—achieving AUCs of 0.871 and 0.848 in the training and validation cohorts, respectively, significantly surpassing PSI (0.738) and CURB-65 (0.714). Calibration curves and H-L tests confirmed high agreement between predicted and observed probabilities; DCA demonstrated consistently superior net clinical benefit over traditional scores across threshold probabilities of 0–0.6; NRI and IDI analyses further quantified significant incremental predictive value.

The PSI and CURB-65 frameworks are primarily anchored in demographic characteristics and acute physiological derangement parameters, and they lack effective capture of infection-induced systemic inflammatory intensity, host nutritional-immunological reserve, and metabolic stress—key pathophysiological dimensions of pneumonia. The inflammatory-nutritional-metabolic composite indices incorporated in the present study provide a systematic complement to these limitations at the biological level. MHR demonstrated the strongest independent predictive performance among all indices (OR = 2.063) and ranked highest in the Random Forest variable importance analysis (mean decrease in Gini = 8.5). Its pathophysiological basis lies in the overactivation of monocytes driving a pro-inflammatory cytokine cascade, while HDL-C is substantially consumed during severe infection, losing its endogenous protective functions of neutralizing bacterial lipopolysaccharide and suppressing inflammatory signaling pathways (26). An elevated MHR thus serves as a composite signal of high inflammatory burden concurrent with depleted anti-inflammatory capacity, providing a well-grounded mechanistic rationale for its independent prognostic value in pneumonia. The HALP index (a protective factor; OR = 0.965) integrates hemoglobin (reflecting oxygen delivery and chronic nutritional status), albumin (a negative acute-phase reactant), lymphocyte count (cellular immune reserve), and platelet count (thrombo-inflammatory signaling) into a single continuous variable. In the present study, the HALP index in the adverse outcome group (14.28) was only 43.8% of that in the good-prognosis group (32.64) (*p* < 0.001), reflecting severe depletion of overall immune-nutritional reserves at hospital admission (27). The TyG index (OR = 1.619), as a surrogate marker of insulin resistance, has been primarily studied in metabolic and cardiovascular diseases; the present study is among the first to systematically demonstrate its independent prognostic value in a disease-specific pneumonia cohort (28). Mechanistically, impaired cellular glucose utilization due to insulin resistance may compromise the phagocytic and bactericidal functions of neutrophils and macrophages, while hypertriglyceridemia is closely associated with impaired endotoxin clearance and endothelial injury, collectively amplifying infection-induced organ damage risk (29, 30). Notably, correlation analysis demonstrated that absolute pairwise correlation coefficients among the three core indices did not exceed 0.08, with the TyG index showing correlations as low as 0.01 with the remaining indices, confirming that each captures mutually independent pathophysiological information and that their combined use does not introduce severe multicollinearity—providing a statistical explanation for why the multidimensional integration strategy outperforms any single index (31).

The traditional clinical parameters incorporated in the model (SpO₂, PaO₂/FiO₂, PCT, CURB-65, and age) and the inflammatory-nutritional-metabolic composite indices are not substitutes for one another but operate synergistically. The former directly reflect pulmonary gas exchange impairment, bacterial infectious burden, and physiological reserve decline—the most immediate physiological warning signals for imminent IMV—while the composite indices capture deeper host immune-metabolic states. The fusion of these two information streams enables the model to simultaneously assess both “current disease severity” and “host coping capacity.” Multicollinearity assessment (all VIF < 3.0) further confirms that each of the nine independent predictors contributes irreplaceable predictive information. Additionally, the comparable AUCs of Random Forest and XGBoost (0.868 and 0.862) with the nomogram (all *p* > 0.05) in the validation cohort indicate that, with adequate feature engineering, an interpretable linear logistic regression model can achieve predictive performance on par with complex nonlinear machine learning models (32), while retaining irreplaceable practical advantages for clinical implementation and dissemination.

Subgroup analyses support the cross-population robustness of the prediction model, with consistent predictive performance across age, sex, and diabetes subgroups (all P for interaction >0.15). A statistically significant interaction was observed for PSI risk-class subgroups (P for interaction = 0.045), with the OR among high-risk patients (PSI class IV–V; 2.25) exceeding that among low-to-moderate-risk patients (1.80), suggesting that inflammatory-nutritional-metabolic composite indices may achieve more refined secondary risk stratification beyond conventional scores among patients already identified as more severely ill, helping clinicians identify the highest-risk subgroup within high-risk populations who require immediate escalation of treatment intensity (33). Risk reclassification visualization revealed the clinical meaning of the new model’s incremental value: while maintaining 100% capture of actual adverse-outcome patients (zero missed cases), the nomogram successfully downgraded 19.3% of good-prognosis patients falsely overclassified by PSI to low-risk, effectively reducing unnecessary escalation of care and wasteful consumption of medical resources without sacrificing sensitivity—a feature of direct decision-making value in settings where ICU beds are limited.

The THM simplified integer score (AUC = 0.804), constructed from the three highest-weighted inflammatory-nutritional-metabolic indices, represents a key translational effort in this study. The THM score significantly outperforms both PSI (0.738) and CURB-65 (0.714), incurring only approximately 0.04 AUC reduction compared with the full nomogram, yet substantially improving bedside operability. The significant gradient trend across low-risk (4.7%), intermediate-risk (16.6%), and high-risk (25.2%) groups (Cochran-Armitage trend test *p* < 0.001), with an OR difference of 6.72-fold between high-risk and low-risk groups, provides a quantitative basis for clinical triage. NRI/IDI analyses further confirmed statistically significant incremental value over traditional scores (all *p* < 0.001).

Several limitations of this study warrant acknowledgment. First, the single-center retrospective design and potential selection bias inherent to institution-specific clinical practices limit the generalizability of the model; multicenter prospective external validation represents the primary objective for future research. Moreover, the composite endpoint of ICU admission and/or IMV is intrinsically susceptible to institutional heterogeneity, as ICU triage thresholds, bed availability, and local resource allocation policies may influence outcome ascertainment independently of true physiological severity; this non-clinical variation could attenuate model transportability, and IMV-only or organ-failure–anchored endpoints should be considered as supplementary outcomes in future external validation. Second, the relatively modest size of the validation cohort (*n* = 252) may affect the precision of some subgroup estimates. Third, the absence of systematic microbiological data precluded etiology-stratified analyses; because bacterial, viral, and atypical pathogens elicit divergent host inflammatory and metabolic signatures—differentially modulating NLR, MHR, and TyG kinetics—the discriminative performance of the composite indices may vary across pathogen subgroups, and pathogen-stratified validation in pathogen-confirmed prospective cohorts is therefore warranted before generalizing the model across the full etiological spectrum of pneumonia. Furthermore, the THM score cutoff values were determined based on training set data, and their stability requires further validation in larger independent cohorts. Notwithstanding these limitations, this study systematically demonstrates the clinical value of incorporating inflammatory-nutritional-metabolic composite indices into the pneumonia prognostic evaluation framework.

In conclusion, the nomogram model and THM simplified score developed and validated in this study, through systematic integration of inflammatory-nutritional-metabolic composite indices, achieve substantial advances over conventional PSI and CURB-65 in predicting short-term adverse outcomes among hospitalized pneumonia patients. These tools may provide more accurate quantitative support for emergency triage and individualized clinical decision-making, and their real-world clinical value warrants further verification in future multicenter prospective studies.

## Data Availability

The raw data supporting the conclusions of this article will be made available by the authors, without undue reservation.
