# Supplementary material for: Development and validation of a nomogram integrating inflammatory-nutritional-metabolic composite indices for predicting short-term adverse outcomes in hospitalized patients with pneumonia
Source: Front Med (Lausanne). 2026 Jun 1;13:1846682. doi: 10.3389/fmed.2026.1846682 (PMC13265524; doi:10.3389/fmed.2026.1846682)
Supplement: Supplementary file 1 [file Table_1.docx]

**Supplementary Table 1. LASSO regression variable selection results (training set, n=587)**

| **Candidate variable** | **Selected** | **Coefficient at λ.min** | **Reason** | **Direction** |
| --- | --- | --- | --- | --- |
| Age | ✓ Retained | 0.018 | Non-zero after penalization | Positive |
| Sex | — Excluded | 0 | Shrunk to zero | — |
| BMI | — Excluded | 0 | Shrunk to zero | — |
| SpO₂ | ✓ Retained | −0.071 | Non-zero after penalization | Negative |
| Heart rate | — Excluded | 0 | Shrunk to zero | — |
| Respiratory rate | — Excluded | 0 | Shrunk to zero | — |
| Altered consciousness | — Excluded | 0 | Shrunk to zero | — |
| COPD | ✓ Retained | 0.482 | Non-zero after penalization | Positive |
| Diabetes mellitus | ✓ Retained | 0.364 | Non-zero after penalization | Positive |
| Heart failure | — Excluded | 0 | Shrunk to zero | — |
| Malignancy | — Excluded | 0 | Shrunk to zero | — |
| NLR | ✓ Retained | 0.054 | Non-zero after penalization | Positive |
| PLR | — Excluded | 0 | Shrunk to zero | — |
| LMR | — Excluded | 0 | Shrunk to zero | — |
| ELR | — Excluded | 0 | Shrunk to zero | — |
| HALP index | ✓ Retained | −0.032 | Non-zero after penalization | Negative |
| TyG index | ✓ Retained | 0.446 | Non-zero after penalization | Positive |
| MHR | ✓ Retained | 0.658 | Non-zero after penalization | Positive |
| Procalcitonin | ✓ Retained | 0.038 | Non-zero after penalization | Positive |
| Albumin | — Excluded | 0 | Shrunk to zero | — |
| PaO₂/FiO₂ | ✓ Retained | −0.005 | Non-zero after penalization | Negative |
| CURB-65 score | ✓ Retained | 0.336 | Non-zero after penalization | Positive |
| D-dimer | — Excluded | 0 | Shrunk to zero | — |
| NT-proBNP | — Excluded | 0 | Shrunk to zero | — |
| Creatinine | — Excluded | 0 | Shrunk to zero | — |

Note: 25 candidate variables were included; optimal λ.min=0.028 was determined by 10-fold cross-validation, yielding 11 non-zero variables. COPD (P=0.069) and diabetes mellitus (P=0.113) did not reach significance in the multivariable model and were excluded; the final nomogram was constructed using the remaining 9 variables. A positive coefficient indicates the variable is associated with increased risk of adverse outcome; negative coefficient indicates association with decreased risk.

**Supplementary Table 2. Construction and risk stratification validation of the THM composite score**

| **Component / Stratum** | **Threshold or score range** | **Direction or n** | **Score or event rate** | **Univariable AUC or OR (95% CI)** | **P value** |
| --- | --- | --- | --- | --- | --- |
| **Part Ⅰ. THM score components and assigned weights** | | | | | |
| TyG index | ≥9.0 | ↑ Elevated | 2 points | 0.638 (0.594–0.682) | — |
| MHR | ≥0.52 | ↑ Elevated | 2 points | 0.724 (0.682–0.766) | — |
| HALP index | ≤20 | ↓ Decreased | 2 points | 0.782 (0.744–0.820) | — |
| Total score range: 0–6 | — | — | — | Overall AUC: 0.804 (0.763–0.845) | — |
| **Part Ⅱ. THM score risk strata and adverse outcome rates** | | | | | |
| Low risk (0–2 points) | 0–2 | 148 | 7 (4.7%) | Reference | — |
| Intermediate risk (3–4 points) | 3–4 | 386 | 64 (16.6%) | 3.96 (1.74–9.02) | **0.001** |
| High risk (5–6 points) | 5–6 | 305 | 77 (25.2%) | 6.72 (2.96–15.28) | **<0.001** |

Note: Thresholds were determined by the Youden index (maximizing sensitivity + specificity − 1). THM score = TyG score + MHR score + HALP score (total: 0–6 points). The β-coefficient ratio for the three predictors (TyG: 0.482, MHR: 0.724, HALP: 0.036) approximated 1.3:2.0:1.0; to facilitate bedside use, weights were simplified to 2 points each. ORs for intermediate- and high-risk groups are referenced to the low-risk group, derived from multivariable logistic regression. Cochran-Armitage trend test P<0.001. AUC = area under the receiver operating characteristic curve.

**Supplementary Table 3. Comparison of secondary outcomes between groups (n=839)**

| **Secondary outcome** | **Adverse outcome group (n=148)** | **Good outcome group (n=691)** | **P value** |
| --- | --- | --- | --- |
| Total length of hospital stay (days), M (IQR) | 15.2 (10.4–22.6) | 9.1 (6.2–13.4) | **<0.001** |
| **In-hospital complications** | | | |
| Sepsis, n (%) | 88 (59.5%) | 83 (12.0%) | **<0.001** |
| Septic shock, n (%) | 43 (29.1%) | 18 (2.6%) | **<0.001** |
| Acute respiratory distress syndrome, n (%) | 69 (46.6%) | 23 (3.3%) | **<0.001** |
| Acute kidney injury, n (%) | 55 (37.2%) | 62 (9.0%) | **<0.001** |
| 30-day readmission rate, n (%) | 29 (19.6%) | 67 (9.7%) | **0.001** |

Note: Length of stay compared by Mann-Whitney U test; categorical variables by χ² test. Adverse outcome group (n=148): in-hospital ICU admission and/or invasive mechanical ventilation. Good outcome group (n=691): neither event occurred.
